# Tetra­kis(μ_2_-2-phen­oxy­propionato)-κ^3^ *O*,*O*′:*O*′;κ^3^ *O*:*O*,*O*′;κ^4^ *O*:*O*′-bis­[(1,10-phenanthroline-κ^2^ *N*,*N*′)(2-phen­oxy­propionato-κ^2^ *O*,*O*′)cerium(III)]

**DOI:** 10.1107/S1600536811036129

**Published:** 2011-09-14

**Authors:** Jin-Bei Shen, Jia-Lu Liu, Guo-Liang Zhao

**Affiliations:** a College of Chemistry and Life Science, Zhejiang Normal University, Jinhua 321004, Zhejiang, People’s Republic of China; b Zhejiang Normal University Xingzhi College, Jinhua, Zhejiang 321004, People’s Republic of China

## Abstract

In the centrosymmetric binuclear title complex, [Ce_2_(C_9_H_9_O_3_)_6_(C_12_H_8_N_2_)_2_], the two Ce^III^ ions are linked by four 2-phen­oxy­propionate groups in bi- and tridentate bridging modes. Each Ce^III^ ion is nine-coordinated by one 1,10-phenanthroline mol­ecule, two O atoms from a chelating carboxyl­ate, two O atoms derived from a μ_3_-carboxylate and two O atoms derived from two μ_2_-carboxylate ligands in a distorted CeN_2_O_7_ monocapped square-anti­prismatic geometry.

## Related literature

For background to phen­oxy­alkanoic acids, see: Markus & Buser (1997 ▶). For a related Ce complex, see: Fu *et al.* (2007 ▶) and for a related La complex, see: Li *et al.* (2010 ▶). For isotypic structures, see: Shen *et al.* (2011*a* ▶) for Tb; Shen *et al.* (2011*b* ▶) for Pr; Shen *et al.* (2011*c* ▶) for Dy; Shen *et al.* (2011*d* ▶) for La; Shen *et al.* (2011*e* ▶) for Ho; Shen *et al.* (2011*f* ▶) for Gd.
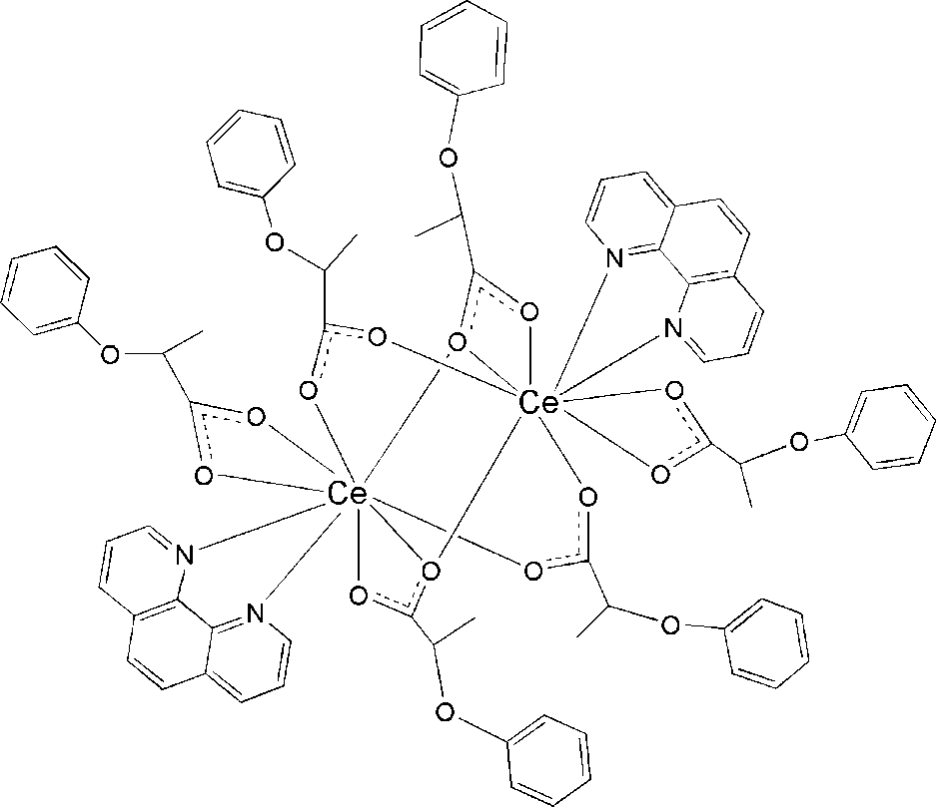


## Experimental

### 

#### Crystal data

[Ce_2_(C_9_H_9_O_3_)_6_(C_12_H_8_N_2_)_2_]
*M* *_r_* = 1631.62
Monoclinic, 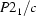

*a* = 11.5137 (2) Å
*b* = 25.9311 (6) Å
*c* = 13.9620 (3) Å
β = 120.149 (1)°
*V* = 3604.62 (13) Å^3^
*Z* = 2
Mo *K*α radiation
μ = 1.32 mm^−1^
*T* = 296 K
0.30 × 0.18 × 0.13 mm

#### Data collection

Bruker APEXII CCD diffractometer
Absorption correction: multi-scan (*SADABS*; Sheldrick, 1996 ▶) *T*_min_ = 0.747, *T*_max_ = 0.848
26086 measured reflections
6351 independent reflections
5383 reflections with *I* > 2σ(*I*)
*R*_int_ = 0.029

#### Refinement

*R*[*F* ^2^ > 2σ(*F* ^2^)] = 0.032
*wR*(*F* ^2^) = 0.060
*S* = 1.10
6351 reflections
461 parameters
234 restraints
H-atom parameters constrained
Δρ_max_ = 0.67 e Å^−3^
Δρ_min_ = −0.35 e Å^−3^

### 

Data collection: *APEX2* (Bruker, 2006 ▶); cell refinement: *SAINT* (Bruker, 2006 ▶); data reduction: *SAINT*; program(s) used to solve structure: *SHELXS97* (Sheldrick, 2008 ▶); program(s) used to refine structure: *SHELXL97* (Sheldrick, 2008 ▶); molecular graphics: *XP* in *SHELXTL* (Sheldrick, 2008 ▶) and *DIAMOND* (Brandenburg, 2006 ▶); software used to prepare material for publication: *SHELXL97*.

## Supplementary Material

Crystal structure: contains datablock(s) I. DOI: 10.1107/S1600536811036129/wm2527sup1.cif

Structure factors: contains datablock(s) I. DOI: 10.1107/S1600536811036129/wm2527Isup2.hkl

Additional supplementary materials:  crystallographic information; 3D view; checkCIF report

## Figures and Tables

**Table 1:** Selected bond lengths (Å)

Ce1—O8^i^	2.4377 (19)
Ce1—O2^i^	2.448 (2)
Ce1—O3	2.483 (2)
Ce1—O5	2.531 (2)
Ce1—O4	2.549 (2)
Ce1—O7	2.5699 (19)
Ce1—N2	2.643 (2)
Ce1—N1	2.691 (2)
Ce1—O8	2.694 (2)

Symmetry code: (i) 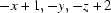.

**Fig. 1.**
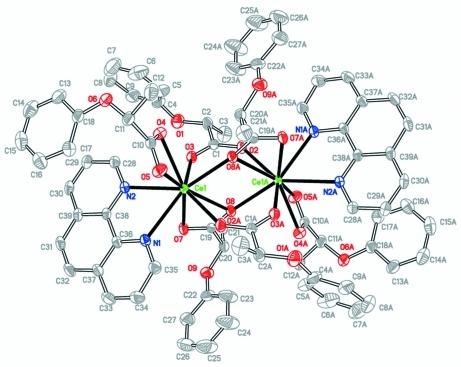
The molecular structure of the title complex, showing the atom-labelling scheme. Displacement ellipsoids are drawn at the 30% probability level.

**Fig. 2.**
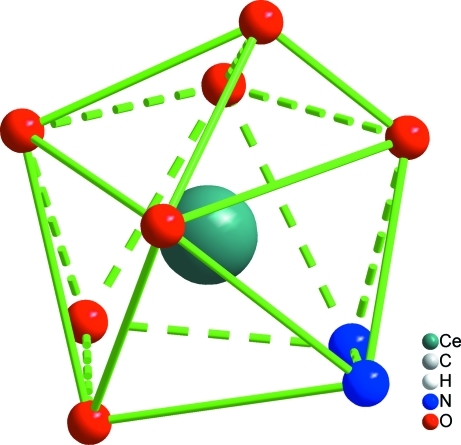
The coordination environment of the Ce(III) atom.

## supplementary crystallographic information

### Comment

The group of phenoxyalkanoic acids includes a considerable number of important herbicides. The desired biological activity is largely dependent on the length of the carbon chain of the alkanoic acid, the nature of the phenoxy group, and the position of its attachment to the carbon chain (Markus & Buser, 1997). The structures of 2-phenoxypropionic acid (H*L*) complexes coupled with their special functionality catched our interest. Here, we describe the Ce^III^ title complex, (I).

The structure of complex (I) is shown in Fig. 1 and the coordination environment of Ce(III) is shown in Fig. 2. The dimeric title compound (I) is centrosymmetric and is comprised of six *L* anions and two phenanthroline ligands. The two Ce(III) ions are linked together by four *L* groups through their bi- and tri-dentate bridging modes, forming a dimeric unit. The distance between two La(III) ions in the dimeric unit is 4.1025 (3) Å. Each Ce(III) ion is coordinated by nine atoms, five of which are oxygen atoms from the bridging carboxylates, two oxygen atoms from the bidentate chelating carboxylate group, and two nitrogen atoms from a 1,10-phenanthroline molecule. The Ce(III) ion adopts a distorted monocapped square antiprisatic geometry (Fig. 2). The Ce—O distances are within the range 2.4377 (19)–2.694 (2) Å, and the Ce—N distances rang from 2.643 (2)–2.691 (2) Å, all of which are within the range of those of other nine-coordinated Ce^III^ complexes with carboxylic donor ligands and 1,10-phenanthroline (Fu *et al.*, 2007) (Table 1).

For background to phenoxyalkanoic acids, see: Markus & Buser (1997). For a related La complex, see: Li *et al.* (2010). For isotypic structures, see: for Tb (Shen *et al.*, 2011*a*), for Pr (Shen *et al.*, 2011*b*), for Dy (Shen *et al.*, 2011*c*), for La (Shen *et al.*, 2011*d*), for Ho (Shen *et al.*, 2011*e*), for Gd (Shen *et al.*, 2011*f*).

### Experimental

Reagents and solvents used were of commercially available quality and without purified before use. 2-Phenoxypropionic acid (1.5 mmol), Ce(NO_3_)_3_^.^6H_2_O (0.5 mmol) and 1,10-phenanthroline (0.5 mmol) were dissolved in 20 ml enthanol, then 10 ml water were added to the above solution. The mixed solution was stirred for 12 h at room temperature. Finally, the deposit was filtered off and the colourless solution was kept in the open air. Colourless crystals were obtained after several days.

### Refinement

The structure was solved by direct methods and successive Fourier difference synthesis. The H atoms bonded to C and N atoms were positioned geometrically and refined using a riding model [aliphatic C—H =0.96 Å (*U*_iso_(H) = 1.5*U*_eq_(C)), aromatic C—H = 0.93 Å (*U*_iso_(H) = 1.2*U*_eq_(C))].

### Crystal data

**Table:**

[Ce_2_(C_9_H_9_O_3_)_6_(C_12_H_8_N_2_)_2_]	*F*(000) = 1652
*M**_r_* = 1631.62	*D*_x_ = 1.503 Mg m^−^^3^
Monoclinic, *P*2_1_/*c*	Mo *K*α radiation, λ = 0.71073 Å
Hall symbol: -P 2ybc	Cell parameters from 9702 reflections
*a* = 11.5137 (2) Å	θ = 1.9–25.0°
*b* = 25.9311 (6) Å	µ = 1.32 mm^−^^1^
*c* = 13.9620 (3) Å	*T* = 296 K
β = 120.149 (1)°	Block, colourless
*V* = 3604.62 (13) Å^3^	0.30 × 0.18 × 0.13 mm
*Z* = 2	

### Data collection

**Table:**

Bruker APEXII CCD diffractometer	6351 independent reflections
Radiation source: fine-focus sealed tube	5383 reflections with *I* > 2σ(*I*)
graphite	*R*_int_ = 0.029
phi and ω scans	θ_max_ = 25.0°, θ_min_ = 1.9°
Absorption correction: multi-scan (*SADABS*; Sheldrick, 1996)	*h* = −13→13
*T*_min_ = 0.747, *T*_max_ = 0.848	*k* = −30→26
26086 measured reflections	*l* = −12→16

### Refinement

**Table:**

Refinement on *F*^2^	Primary atom site location: structure-invariant direct methods
Least-squares matrix: full	Secondary atom site location: difference Fourier map
*R*[*F*^2^ > 2σ(*F*^2^)] = 0.032	Hydrogen site location: inferred from neighbouring sites
*wR*(*F*^2^) = 0.060	H-atom parameters constrained
*S* = 1.10	*w* = 1/[σ^2^(*F*_o_^2^) + (0.0098*P*)^2^ + 3.9343*P*] where *P* = (*F*_o_^2^ + 2*F*_c_^2^)/3
6351 reflections	(Δ/σ)_max_ = 0.001
461 parameters	Δρ_max_ = 0.67 e Å^−^^3^
234 restraints	Δρ_min_ = −0.35 e Å^−^^3^

### Special details

**Table:**

Geometry. All e.s.d.'s (except the e.s.d. in the dihedral angle between two l.s. planes) are estimated using the full covariance matrix. The cell e.s.d.'s are taken into account individually in the estimation of e.s.d.'s in distances, angles and torsion angles; correlations between e.s.d.'s in cell parameters are only used when they are defined by crystal symmetry. An approximate (isotropic) treatment of cell e.s.d.'s is used for estimating e.s.d.'s involving l.s. planes.
Refinement. Refinement of *F*^2^ against ALL reflections. The weighted *R*-factor *wR* and goodness of fit *S* are based on *F*^2^, conventional *R*-factors *R* are based on *F*, with *F* set to zero for negative *F*^2^. The threshold expression of *F*^2^ > σ(*F*^2^) is used only for calculating *R*-factors(gt) *etc*. and is not relevant to the choice of reflections for refinement. *R*-factors based on *F*^2^ are statistically about twice as large as those based on *F*, and *R*- factors based on ALL data will be even larger.

### Fractional atomic coordinates and isotropic or equivalent isotropic displacement parameters (Å^2^)

**Table:**

	*x*	*y*	*z*	*U*_iso_*/*U*_eq_	
Ce1	0.454529 (15)	−0.002855 (6)	0.837971 (13)	0.03007 (6)	
C1	0.3410 (3)	−0.08904 (12)	0.9639 (3)	0.0374 (7)	
C2	0.2597 (3)	−0.13715 (14)	0.9555 (3)	0.0478 (9)	
H2A	0.3232	−0.1649	0.9966	0.057*	
C3	0.1761 (4)	−0.12764 (17)	1.0088 (4)	0.0729 (12)	
H3A	0.1256	−0.1581	1.0029	0.109*	
H3B	0.1155	−0.0995	0.9720	0.109*	
H3C	0.2338	−0.1192	1.0855	0.109*	
C4	0.2261 (4)	−0.18348 (14)	0.7942 (3)	0.0583 (10)	
C5	0.3567 (4)	−0.18465 (15)	0.8199 (4)	0.0686 (12)	
H5A	0.4220	−0.1670	0.8815	0.082*	
C6	0.3915 (6)	−0.21221 (19)	0.7536 (6)	0.1023 (18)	
H6A	0.4806	−0.2133	0.7706	0.123*	
C7	0.2961 (9)	−0.2377 (2)	0.6639 (6)	0.125 (2)	
H7A	0.3201	−0.2559	0.6191	0.150*	
C8	0.1657 (8)	−0.2371 (2)	0.6384 (5)	0.116 (2)	
H8A	0.1013	−0.2552	0.5772	0.139*	
C9	0.1294 (5)	−0.20981 (16)	0.7028 (4)	0.0821 (14)	
H9A	0.0400	−0.2090	0.6852	0.098*	
C10	0.6081 (3)	−0.06332 (14)	0.7670 (3)	0.0399 (8)	
C11	0.6786 (3)	−0.09362 (14)	0.7173 (3)	0.0515 (9)	
H11A	0.6990	−0.0706	0.6720	0.062*	
C12	0.8082 (4)	−0.11664 (17)	0.8094 (3)	0.0729 (13)	
H12A	0.8522	−0.1357	0.7777	0.109*	
H12B	0.7882	−0.1393	0.8535	0.109*	
H12C	0.8661	−0.0895	0.8551	0.109*	
C13	0.4214 (4)	−0.16838 (16)	0.4901 (3)	0.0633 (11)	
H13A	0.4448	−0.2008	0.5231	0.076*	
C14	0.3159 (4)	−0.16336 (19)	0.3840 (4)	0.0755 (13)	
H14A	0.2681	−0.1924	0.3453	0.091*	
C15	0.2803 (4)	−0.1160 (2)	0.3347 (3)	0.0738 (13)	
H15A	0.2090	−0.1127	0.2626	0.089*	
C16	0.3503 (4)	−0.07341 (18)	0.3922 (3)	0.0683 (12)	
H16A	0.3262	−0.0412	0.3587	0.082*	
C17	0.4570 (4)	−0.07747 (16)	0.4999 (3)	0.0567 (10)	
H17A	0.5033	−0.0482	0.5391	0.068*	
C18	0.4929 (3)	−0.12559 (15)	0.5477 (3)	0.0500 (9)	
C19	0.2664 (3)	0.04852 (11)	0.8978 (3)	0.0323 (7)	
C20	0.1689 (3)	0.07364 (13)	0.9267 (3)	0.0417 (8)	
H20A	0.2187	0.0900	0.9997	0.050*	
C21	0.0748 (4)	0.03318 (15)	0.9278 (3)	0.0596 (10)	
H21A	0.0131	0.0491	0.9461	0.089*	
H21B	0.1259	0.0073	0.9820	0.089*	
H21C	0.0256	0.0175	0.8560	0.089*	
C22	0.1500 (3)	0.15388 (14)	0.8359 (3)	0.0544 (10)	
C23	0.2812 (4)	0.16723 (16)	0.9091 (4)	0.0943 (17)	
H23A	0.3347	0.1461	0.9693	0.113*	
C24	0.3328 (5)	0.21212 (19)	0.8924 (5)	0.123 (2)	
H24A	0.4208	0.2215	0.9429	0.147*	
C25	0.2580 (6)	0.24283 (19)	0.8041 (5)	0.110 (2)	
H25A	0.2947	0.2727	0.7934	0.132*	
C26	0.1280 (6)	0.22961 (18)	0.7308 (4)	0.0967 (17)	
H26A	0.0758	0.2506	0.6700	0.116*	
C27	0.0741 (4)	0.18526 (16)	0.7468 (4)	0.0731 (12)	
H27A	−0.0146	0.1765	0.6968	0.088*	
C28	0.2365 (3)	−0.07862 (13)	0.6103 (3)	0.0436 (8)	
H28A	0.2803	−0.1034	0.6651	0.052*	
C29	0.1295 (3)	−0.09414 (14)	0.5082 (3)	0.0488 (9)	
H29A	0.1039	−0.1286	0.4952	0.059*	
C30	0.0633 (3)	−0.05827 (14)	0.4280 (3)	0.0472 (9)	
H30A	−0.0096	−0.0679	0.3602	0.057*	
C31	0.0403 (3)	0.03314 (15)	0.3681 (3)	0.0497 (9)	
H31A	−0.0338	0.0252	0.2995	0.060*	
C32	0.0839 (3)	0.08178 (15)	0.3900 (3)	0.0528 (9)	
H32A	0.0386	0.1071	0.3368	0.063*	
C33	0.2500 (4)	0.14576 (15)	0.5189 (3)	0.0562 (10)	
H33A	0.2098	0.1719	0.4669	0.067*	
C34	0.3588 (4)	0.15641 (14)	0.6195 (3)	0.0603 (10)	
H34A	0.3938	0.1896	0.6368	0.072*	
C35	0.4167 (3)	0.11648 (13)	0.6961 (3)	0.0489 (9)	
H35A	0.4909	0.1241	0.7647	0.059*	
C36	0.2641 (3)	0.05794 (12)	0.5758 (2)	0.0352 (7)	
C37	0.1985 (3)	0.09594 (13)	0.4935 (3)	0.0425 (8)	
C38	0.2155 (3)	0.00588 (12)	0.5525 (2)	0.0343 (7)	
C39	0.1048 (3)	−0.00695 (14)	0.4475 (2)	0.0412 (8)	
N1	0.3723 (2)	0.06856 (10)	0.6769 (2)	0.0371 (6)	
N2	0.2785 (2)	−0.03027 (10)	0.6328 (2)	0.0371 (6)	
O1	0.1737 (2)	−0.15482 (10)	0.8471 (2)	0.0636 (7)	
O9	0.0877 (2)	0.11100 (9)	0.8460 (2)	0.0515 (6)	
O5	0.6289 (2)	−0.01570 (9)	0.7804 (2)	0.0521 (6)	
O4	0.5362 (2)	−0.08674 (8)	0.79660 (18)	0.0450 (6)	
O7	0.23777 (19)	0.04315 (8)	0.80004 (17)	0.0396 (5)	
O3	0.3308 (2)	−0.06985 (8)	0.87816 (18)	0.0412 (5)	
O2	0.4146 (2)	−0.07312 (8)	1.06170 (18)	0.0463 (6)	
O8	0.37595 (19)	0.03220 (8)	0.97824 (16)	0.0393 (5)	
O6	0.6000 (2)	−0.13560 (9)	0.6518 (2)	0.0603 (7)	

### Atomic displacement parameters (Å^2^)

**Table:**

	*U*^11^	*U*^22^	*U*^33^	*U*^12^	*U*^13^	*U*^23^
Ce1	0.02866 (8)	0.03356 (10)	0.02299 (9)	0.00020 (8)	0.00926 (7)	−0.00084 (8)
C1	0.0353 (16)	0.0360 (18)	0.036 (2)	−0.0011 (13)	0.0147 (15)	−0.0013 (15)
C2	0.0446 (18)	0.052 (2)	0.035 (2)	−0.0136 (16)	0.0118 (16)	−0.0008 (16)
C3	0.069 (3)	0.086 (3)	0.080 (3)	−0.022 (2)	0.049 (2)	−0.004 (3)
C4	0.079 (3)	0.035 (2)	0.064 (3)	−0.0041 (19)	0.038 (2)	−0.0057 (19)
C5	0.080 (3)	0.045 (2)	0.083 (3)	−0.001 (2)	0.043 (3)	−0.008 (2)
C6	0.136 (5)	0.061 (3)	0.150 (5)	0.003 (3)	0.103 (5)	−0.001 (3)
C7	0.215 (8)	0.079 (4)	0.120 (6)	−0.011 (5)	0.114 (6)	−0.023 (4)
C8	0.177 (6)	0.074 (4)	0.064 (4)	−0.010 (4)	0.036 (4)	−0.023 (3)
C9	0.091 (3)	0.050 (3)	0.073 (3)	−0.005 (2)	0.018 (3)	−0.005 (2)
C10	0.0362 (16)	0.049 (2)	0.0290 (18)	0.0052 (15)	0.0120 (14)	−0.0037 (15)
C11	0.050 (2)	0.058 (2)	0.044 (2)	0.0087 (17)	0.0217 (17)	−0.0092 (18)
C12	0.054 (2)	0.101 (3)	0.053 (3)	0.027 (2)	0.0189 (19)	−0.015 (2)
C13	0.067 (2)	0.057 (3)	0.065 (3)	0.002 (2)	0.032 (2)	−0.015 (2)
C14	0.061 (3)	0.085 (3)	0.074 (3)	−0.010 (2)	0.029 (2)	−0.032 (3)
C15	0.056 (2)	0.118 (4)	0.043 (3)	0.002 (3)	0.022 (2)	−0.006 (3)
C16	0.065 (3)	0.087 (3)	0.055 (3)	0.009 (2)	0.032 (2)	0.016 (2)
C17	0.059 (2)	0.061 (3)	0.051 (2)	−0.0012 (19)	0.029 (2)	−0.001 (2)
C18	0.053 (2)	0.057 (2)	0.042 (2)	0.0091 (17)	0.0253 (18)	−0.0063 (18)
C19	0.0314 (15)	0.0313 (17)	0.0315 (18)	−0.0006 (12)	0.0138 (14)	0.0000 (14)
C20	0.0411 (17)	0.049 (2)	0.0335 (18)	0.0064 (15)	0.0179 (15)	−0.0003 (16)
C21	0.056 (2)	0.073 (3)	0.067 (3)	−0.0010 (19)	0.044 (2)	0.001 (2)
C22	0.045 (2)	0.039 (2)	0.068 (3)	0.0055 (16)	0.0203 (19)	−0.0048 (19)
C23	0.064 (3)	0.051 (3)	0.109 (4)	−0.011 (2)	0.000 (3)	0.018 (3)
C24	0.083 (3)	0.064 (3)	0.156 (5)	−0.018 (3)	0.011 (3)	0.025 (3)
C25	0.100 (4)	0.057 (3)	0.146 (5)	−0.014 (3)	0.041 (4)	0.013 (3)
C26	0.109 (4)	0.059 (3)	0.099 (4)	0.007 (3)	0.034 (3)	0.022 (3)
C27	0.067 (3)	0.062 (3)	0.072 (3)	0.008 (2)	0.021 (2)	0.003 (2)
C28	0.0463 (18)	0.043 (2)	0.0354 (19)	−0.0032 (15)	0.0163 (15)	−0.0021 (16)
C29	0.0488 (19)	0.054 (2)	0.039 (2)	−0.0087 (17)	0.0189 (17)	−0.0109 (18)
C30	0.0389 (18)	0.065 (2)	0.0283 (19)	−0.0060 (17)	0.0100 (15)	−0.0135 (18)
C31	0.0396 (18)	0.075 (3)	0.0246 (18)	0.0087 (18)	0.0084 (15)	0.0014 (18)
C32	0.049 (2)	0.067 (3)	0.035 (2)	0.0180 (18)	0.0153 (16)	0.0173 (18)
C33	0.062 (2)	0.053 (2)	0.052 (2)	0.0124 (18)	0.027 (2)	0.0172 (19)
C34	0.071 (2)	0.041 (2)	0.063 (3)	−0.0028 (18)	0.030 (2)	0.0059 (19)
C35	0.050 (2)	0.047 (2)	0.042 (2)	−0.0059 (16)	0.0181 (16)	0.0004 (17)
C36	0.0326 (15)	0.047 (2)	0.0271 (17)	0.0047 (14)	0.0162 (13)	0.0052 (14)
C37	0.0436 (18)	0.052 (2)	0.0339 (19)	0.0112 (16)	0.0210 (15)	0.0099 (16)
C38	0.0311 (14)	0.0468 (19)	0.0263 (16)	0.0024 (14)	0.0154 (12)	−0.0018 (15)
C39	0.0347 (15)	0.061 (2)	0.0271 (16)	0.0057 (16)	0.0151 (13)	−0.0024 (16)
N1	0.0359 (13)	0.0422 (16)	0.0290 (15)	−0.0028 (12)	0.0131 (11)	0.0012 (12)
N2	0.0394 (14)	0.0406 (16)	0.0262 (14)	0.0006 (12)	0.0127 (12)	−0.0001 (12)
O1	0.0519 (15)	0.0612 (17)	0.0607 (18)	−0.0119 (13)	0.0157 (13)	−0.0123 (14)
O9	0.0351 (12)	0.0490 (15)	0.0584 (16)	0.0079 (10)	0.0146 (11)	0.0012 (12)
O5	0.0618 (15)	0.0451 (15)	0.0636 (17)	−0.0034 (11)	0.0421 (13)	−0.0073 (12)
O4	0.0461 (13)	0.0408 (13)	0.0495 (15)	−0.0010 (10)	0.0251 (12)	−0.0048 (11)
O7	0.0348 (11)	0.0533 (14)	0.0253 (12)	0.0073 (10)	0.0111 (9)	−0.0002 (10)
O3	0.0489 (12)	0.0398 (13)	0.0324 (13)	−0.0074 (10)	0.0183 (10)	−0.0026 (10)
O2	0.0541 (13)	0.0455 (14)	0.0312 (13)	−0.0144 (11)	0.0155 (11)	−0.0045 (11)
O8	0.0358 (11)	0.0452 (13)	0.0278 (12)	0.0073 (10)	0.0092 (9)	0.0001 (10)
O6	0.0694 (16)	0.0535 (16)	0.0455 (16)	0.0142 (13)	0.0196 (13)	−0.0097 (12)

### Geometric parameters (Å, °)

**Table:**

Ce1—O8^i^	2.4377 (19)	C17—C18	1.377 (5)
Ce1—O2^i^	2.448 (2)	C17—H17A	0.9300
Ce1—O3	2.483 (2)	C18—O6	1.379 (4)
Ce1—O5	2.531 (2)	C19—O7	1.241 (3)
Ce1—O4	2.549 (2)	C19—O8	1.268 (3)
Ce1—O7	2.5699 (19)	C19—C20	1.517 (4)
Ce1—N2	2.643 (2)	C20—O9	1.423 (4)
Ce1—N1	2.691 (2)	C20—C21	1.514 (5)
Ce1—O8	2.694 (2)	C20—H20A	0.9800
Ce1—C10	2.886 (3)	C21—H21A	0.9600
Ce1—C19	2.997 (3)	C21—H21B	0.9600
Ce1—Ce1^i^	4.1025 (3)	C21—H21C	0.9600
C1—O3	1.247 (4)	C22—O9	1.369 (4)
C1—O2	1.261 (4)	C22—C27	1.371 (5)
C1—C2	1.529 (4)	C22—C23	1.377 (5)
C2—O1	1.406 (4)	C23—C24	1.378 (6)
C2—C3	1.503 (5)	C23—H23A	0.9300
C2—H2A	0.9800	C24—C25	1.353 (7)
C3—H3A	0.9600	C24—H24A	0.9300
C3—H3B	0.9600	C25—C26	1.366 (6)
C3—H3C	0.9600	C25—H25A	0.9300
C4—C5	1.360 (5)	C26—C27	1.377 (6)
C4—C9	1.381 (5)	C26—H26A	0.9300
C4—O1	1.382 (4)	C27—H27A	0.9300
C5—C6	1.377 (6)	C28—N2	1.323 (4)
C5—H5A	0.9300	C28—C29	1.396 (4)
C6—C7	1.353 (8)	C28—H28A	0.9300
C6—H6A	0.9300	C29—C30	1.357 (5)
C7—C8	1.357 (8)	C29—H29A	0.9300
C7—H7A	0.9300	C30—C39	1.394 (5)
C8—C9	1.364 (7)	C30—H30A	0.9300
C8—H8A	0.9300	C31—C32	1.335 (5)
C9—H9A	0.9300	C31—C39	1.426 (5)
C10—O4	1.252 (4)	C31—H31A	0.9300
C10—O5	1.254 (4)	C32—C37	1.431 (5)
C10—C11	1.524 (4)	C32—H32A	0.9300
C11—O6	1.417 (4)	C33—C34	1.360 (5)
C11—C12	1.520 (5)	C33—C37	1.391 (5)
C11—H11A	0.9800	C33—H33A	0.9300
C12—H12A	0.9600	C34—C35	1.394 (5)
C12—H12B	0.9600	C34—H34A	0.9300
C12—H12C	0.9600	C35—N1	1.319 (4)
C13—C14	1.371 (5)	C35—H35A	0.9300
C13—C18	1.375 (5)	C36—N1	1.362 (4)
C13—H13A	0.9300	C36—C37	1.411 (4)
C14—C15	1.367 (6)	C36—C38	1.435 (4)
C14—H14A	0.9300	C38—N2	1.359 (4)
C15—C16	1.364 (6)	C38—C39	1.417 (4)
C15—H15A	0.9300	O2—Ce1^i^	2.448 (2)
C16—C17	1.387 (5)	O8—Ce1^i^	2.4377 (19)
C16—H16A	0.9300		
			
O8^i^—Ce1—O2^i^	73.24 (7)	C11—C12—H12B	109.5
O8^i^—Ce1—O3	77.92 (7)	H12A—C12—H12B	109.5
O2^i^—Ce1—O3	133.77 (7)	C11—C12—H12C	109.5
O8^i^—Ce1—O5	87.12 (7)	H12A—C12—H12C	109.5
O2^i^—Ce1—O5	85.94 (8)	H12B—C12—H12C	109.5
O3—Ce1—O5	128.05 (7)	C14—C13—C18	120.0 (4)
O8^i^—Ce1—O4	77.33 (7)	C14—C13—H13A	120.0
O2^i^—Ce1—O4	128.71 (7)	C18—C13—H13A	120.0
O3—Ce1—O4	76.89 (7)	C15—C14—C13	120.6 (4)
O5—Ce1—O4	51.24 (7)	C15—C14—H14A	119.7
O8^i^—Ce1—O7	122.89 (7)	C13—C14—H14A	119.7
O2^i^—Ce1—O7	89.95 (7)	C16—C15—C14	119.4 (4)
O3—Ce1—O7	76.30 (7)	C16—C15—H15A	120.3
O5—Ce1—O7	146.99 (7)	C14—C15—H15A	120.3
O4—Ce1—O7	141.28 (7)	C15—C16—C17	121.1 (4)
O8^i^—Ce1—N2	146.05 (7)	C15—C16—H16A	119.5
O2^i^—Ce1—N2	138.86 (8)	C17—C16—H16A	119.5
O3—Ce1—N2	80.91 (7)	C18—C17—C16	118.7 (4)
O5—Ce1—N2	85.37 (8)	C18—C17—H17A	120.6
O4—Ce1—N2	72.19 (7)	C16—C17—H17A	120.6
O7—Ce1—N2	76.40 (7)	C13—C18—C17	120.1 (3)
O8^i^—Ce1—N1	148.65 (7)	C13—C18—O6	114.9 (3)
O2^i^—Ce1—N1	77.38 (7)	C17—C18—O6	125.0 (3)
O3—Ce1—N1	132.00 (7)	O7—C19—O8	122.2 (3)
O5—Ce1—N1	80.07 (8)	O7—C19—C20	121.2 (3)
O4—Ce1—N1	114.39 (8)	O8—C19—C20	116.6 (3)
O7—Ce1—N1	67.09 (7)	O7—C19—Ce1	58.21 (15)
N2—Ce1—N1	61.52 (8)	O8—C19—Ce1	64.01 (15)
O8^i^—Ce1—O8	73.97 (7)	C20—C19—Ce1	178.8 (2)
O2^i^—Ce1—O8	69.35 (7)	O9—C20—C21	106.9 (3)
O3—Ce1—O8	68.38 (7)	O9—C20—C19	111.2 (3)
O5—Ce1—O8	152.19 (7)	C21—C20—C19	109.5 (3)
O4—Ce1—O8	138.55 (7)	O9—C20—H20A	109.7
O7—Ce1—O8	49.26 (6)	C21—C20—H20A	109.7
N2—Ce1—O8	121.59 (7)	C19—C20—H20A	109.7
N1—Ce1—O8	105.63 (7)	C20—C21—H21A	109.5
O8^i^—Ce1—C10	83.52 (8)	C20—C21—H21B	109.5
O2^i^—Ce1—C10	109.06 (9)	H21A—C21—H21B	109.5
O3—Ce1—C10	102.57 (9)	C20—C21—H21C	109.5
O5—Ce1—C10	25.71 (8)	H21A—C21—H21C	109.5
O4—Ce1—C10	25.70 (8)	H21B—C21—H21C	109.5
O7—Ce1—C10	151.72 (8)	O9—C22—C27	116.6 (3)
N2—Ce1—C10	75.54 (8)	O9—C22—C23	124.4 (4)
N1—Ce1—C10	96.09 (9)	C27—C22—C23	119.1 (4)
O8—Ce1—C10	156.96 (8)	C22—C23—C24	119.4 (4)
O8^i^—Ce1—C19	98.86 (8)	C22—C23—H23A	120.3
O2^i^—Ce1—C19	78.81 (8)	C24—C23—H23A	120.3
O3—Ce1—C19	70.79 (7)	C25—C24—C23	121.4 (5)
O5—Ce1—C19	161.16 (8)	C25—C24—H24A	119.3
O4—Ce1—C19	147.49 (8)	C23—C24—H24A	119.3
O7—Ce1—C19	24.23 (7)	C24—C25—C26	119.4 (5)
N2—Ce1—C19	98.83 (8)	C24—C25—H25A	120.3
N1—Ce1—C19	85.80 (8)	C26—C25—H25A	120.3
O8—Ce1—C19	25.03 (7)	C25—C26—C27	120.1 (5)
C10—Ce1—C19	172.12 (9)	C25—C26—H26A	119.9
O8^i^—Ce1—Ce1^i^	39.14 (5)	C27—C26—H26A	119.9
O2^i^—Ce1—Ce1^i^	66.24 (5)	C22—C27—C26	120.6 (4)
O3—Ce1—Ce1^i^	68.51 (5)	C22—C27—H27A	119.7
O5—Ce1—Ce1^i^	123.44 (6)	C26—C27—H27A	119.7
O4—Ce1—Ce1^i^	111.22 (5)	N2—C28—C29	123.1 (3)
O7—Ce1—Ce1^i^	83.92 (5)	N2—C28—H28A	118.5
N2—Ce1—Ce1^i^	146.82 (6)	C29—C28—H28A	118.5
N1—Ce1—Ce1^i^	133.19 (6)	C30—C29—C28	119.1 (3)
O8—Ce1—Ce1^i^	34.83 (4)	C30—C29—H29A	120.4
C10—Ce1—Ce1^i^	122.50 (6)	C28—C29—H29A	120.4
C19—Ce1—Ce1^i^	59.76 (6)	C29—C30—C39	119.9 (3)
O3—C1—O2	126.7 (3)	C29—C30—H30A	120.1
O3—C1—C2	119.5 (3)	C39—C30—H30A	120.1
O2—C1—C2	113.8 (3)	C32—C31—C39	121.4 (3)
O1—C2—C3	107.9 (3)	C32—C31—H31A	119.3
O1—C2—C1	115.0 (3)	C39—C31—H31A	119.3
C3—C2—C1	110.4 (3)	C31—C32—C37	121.6 (3)
O1—C2—H2A	107.8	C31—C32—H32A	119.2
C3—C2—H2A	107.8	C37—C32—H32A	119.2
C1—C2—H2A	107.8	C34—C33—C37	120.4 (3)
C2—C3—H3A	109.5	C34—C33—H33A	119.8
C2—C3—H3B	109.5	C37—C33—H33A	119.8
H3A—C3—H3B	109.5	C33—C34—C35	118.6 (3)
C2—C3—H3C	109.5	C33—C34—H34A	120.7
H3A—C3—H3C	109.5	C35—C34—H34A	120.7
H3B—C3—H3C	109.5	N1—C35—C34	123.8 (3)
C5—C4—C9	120.3 (4)	N1—C35—H35A	118.1
C5—C4—O1	126.4 (4)	C34—C35—H35A	118.1
C9—C4—O1	113.2 (4)	N1—C36—C37	122.7 (3)
C4—C5—C6	119.4 (5)	N1—C36—C38	118.3 (3)
C4—C5—H5A	120.3	C37—C36—C38	119.0 (3)
C6—C5—H5A	120.3	C33—C37—C36	117.1 (3)
C7—C6—C5	120.0 (6)	C33—C37—C32	123.7 (3)
C7—C6—H6A	120.0	C36—C37—C32	119.2 (3)
C5—C6—H6A	120.0	N2—C38—C39	121.5 (3)
C6—C7—C8	120.9 (6)	N2—C38—C36	118.4 (3)
C6—C7—H7A	119.6	C39—C38—C36	120.1 (3)
C8—C7—H7A	119.6	C30—C39—C38	117.9 (3)
C7—C8—C9	119.9 (6)	C30—C39—C31	123.4 (3)
C7—C8—H8A	120.1	C38—C39—C31	118.7 (3)
C9—C8—H8A	120.1	C35—N1—C36	117.4 (3)
C8—C9—C4	119.5 (5)	C35—N1—Ce1	122.8 (2)
C8—C9—H9A	120.2	C36—N1—Ce1	118.82 (19)
C4—C9—H9A	120.2	C28—N2—C38	118.5 (3)
O4—C10—O5	122.4 (3)	C28—N2—Ce1	120.1 (2)
O4—C10—C11	119.4 (3)	C38—N2—Ce1	120.66 (19)
O5—C10—C11	118.1 (3)	C4—O1—C2	119.4 (3)
O4—C10—Ce1	61.94 (16)	C22—O9—C20	117.7 (2)
O5—C10—Ce1	61.14 (17)	C10—O5—Ce1	93.1 (2)
C11—C10—Ce1	174.1 (2)	C10—O4—Ce1	92.37 (19)
O6—C11—C12	106.3 (3)	C19—O7—Ce1	97.56 (17)
O6—C11—C10	112.1 (3)	C1—O3—Ce1	135.07 (19)
C12—C11—C10	109.8 (3)	C1—O2—Ce1^i^	140.2 (2)
O6—C11—H11A	109.5	C19—O8—Ce1^i^	161.9 (2)
C12—C11—H11A	109.5	C19—O8—Ce1	90.96 (18)
C10—C11—H11A	109.5	Ce1^i^—O8—Ce1	106.03 (7)
C11—C12—H12A	109.5	C18—O6—C11	118.6 (3)

Symmetry codes: (i) −*x*+1, −*y*, −*z*+2.
